# Emergency Medical Services Time on Scene and Non-Transport: Role of Communication Barriers

**DOI:** 10.5811/westjem.41212

**Published:** 2025-08-20

**Authors:** Elina Kurkurina, Craig Rothenberg, Katherine Couturier, Amelia Breyre, David Yang, Alexander R. Nelson, Alexis Cordone, Arjun K. Venkatesh, Cameron J. Gettel

**Affiliations:** * Quinnipiac University, Frank H. Netter MD School of Medicine, North Haven, Connecticut; † Yale University School of Medicine, Department of Emergency Medicine, New Haven, Connecticut; ‡ Yale University School of Medicine, Center for Outcomes Research and Evaluation, New Haven, Connecticut

## Abstract

**Introduction:**

Clear communication is essential for emergency medical services (EMS) clinicians to assess a situation and make appropriate transport decisions. When barriers are present that impede communication between emergency responders and patients, EMS clinicians report difficulty navigating these encounters. As communication barriers potentially delay definitive care, it remains unclear the amount of time that EMS clinicians spend on scene during these encounters and how often they result in non-transport. In this study we sought to characterize the association between the presence of communication barriers, time spent on scene, and non-transport.

**Methods:**

We conducted an observational analysis using 2022 data from the ESO Data Collaborative, a deidentified national prehospital electronic health record dataset. Encounters were restricted to 9-1-1 responses in which the responding ambulance was first on scene, the patient was alive, ≥ 18 year of age, and able to refuse transport. The primary outcomes were time on scene and non-transport. We used logistic regression models to estimate non-transport by communication barrier (including non-English language preference, speech disability, deaf or hard of hearing, and blind or low vision) and control for key patient and encounter characteristics.

**Results:**

Of 3,477,008 EMS responses, 233,084 (6.7%) resulted in non-transport and 99,263 (2.9%) had a communication barrier identified. Among encounters with a communication barrier identified, EMS clinicians spent more time on scene with patients who were not transported (21.0 minutes) compared to patients who were transported for definitive care (15.9 minutes). Compared to those without an identified barrier, encounters with a patient who had a non-English language preference (odds ratio [OR] 0.51, confidence interval [CI] 0.49–0.53, *P* < .001), patients who had a speech disability (OR 0.36, CI 0.33–0.40, *P* < .001), were deaf or hard of hearing (OR 0.71, CI 0.66–0.76, *P* < .001), or were blind or had low vision (OR 0.80, CI 0.69–0.92, *P* < .001) were less likely to result in non-transport, with non-transport rates of 3.6%, 1.9%, 4.0%, and 4.4% respectively.

**Conclusion:**

Encounters with communication barriers were less likely to end in non-transport. When communication barriers were identified, EMS clinicians spent 32% (5.1 minutes) longer on scene on encounters that resulted in non-transport, showing that EMS clinicians may be dedicating additional time and resources caring for this population.

## INTRODUCTION

Clear and effective communication between patients and clinicians is an important component of safe, high-quality, and informed medical care.^1^ Communication barriers are common, affecting millions of adults in the United States. Recent estimates suggest that 67.8 million individuals in the US speak a language other than English at home,^2^ 37.5 million are deaf or hard of hearing,^3^ three million have a speech disability,^4^ and six million are blind or have low vision.^5^ These communication barriers potentially limit individuals’ ability to effectively communicate with emergency medical services (EMS) clinicians during an acute illness or injury and possibly result in delayed or missed diagnoses and treatment.^6^,^7^ National estimates project an increase in these conditions and limitations, and current efforts are underway to improve equity and accessibility across multiple healthcare settings to better serve these patients.^8^–^12^

Emergency medical services clinicians face numerous challenges when barriers to communication are present, including delayed dispatch and frequent on-scene transport priority changes.^13^ Qualitatively, EMS clinicians describe these situations in which communication barriers are present as high-stress scenarios, express concern about their ability to accurately assess acuity, and acknowledge increased instances of poor rapport with patients.^14^ Meanwhile, quantitative assessments of key metrics, such as time on scene, have varied in their findings. Some studies have found that EMS clinicians spend less time on scene with patients who have communication barriers,^15^,^16^ potentially indicating a desire to quickly transport patients to a facility equipped to meet their acute illness and communication needs.^17^ Conversely, other studies found no difference in transport time for patients with communication barriers.^18^ Despite advancements in EMS practice and the critical role of communication, existing literature offers conflicting and limited insight into how communication barriers impact prehospital care.

Previous studies on communication barriers have focused primarily on language discordance and have been restricted to smaller, regional samples, leaving significant gaps in our understanding of the broader implications of these barriers on EMS operations and patient outcomes. Given the uncertainty in encounters where patients have communication barriers, investigation of on-scene times and non-transport rates is critically important to address potential areas for improvement and/or inequities, especially for less frequently discussed communication barriers such as patients who are deaf or hard of hearing, have speech disabilities, and are blind or have low vision. Excess time on scene and non-transport may delay time-sensitive care,^19^,^20^ especially if the acuity was not properly conveyed,^14^ while simultaneously limiting the unit’s ability to respond to other calls, which can lead to further delays given EMS staffing shortages^21^ and the closure of EMS agencies. To better understand the influence of communication barriers on EMS operations, we used a nationally representative dataset to characterize the association between four common communication barriers (non-English language preference, speech disability, deaf or hard of hearing, and blind or low vision), time on scene, and non-transport.

**Population Health Research Capsule**

What do we already know about this issue?

*Communication barriers in Emergency Medical Services (EMS) encounters may complicate care and delay necessary transport, but time impacts and outcomes are unclear for less frequently studied barriers*.

What was the research question?

*What is the impact of communication barriers on EMS on-scene time and non-transport rates?*

What was the major finding of the study?

*EMS encounters with communication barriers resulted in 5.1 minutes longer on-scene times for non-transported patients and had lower odds of non-transport (OR 0.51, CI 0.49–0.5)*.

How does this improve population health?

*Identifying encounters with communication barriers helps EMS clinicians allocate time, training, and resources appropriately*.

## METHODS

### Study Design and Data Source

We conducted an observational analysis using data from the ESO Data Collaborative. Used previously in numerous prehospital evaluations,^22^–^25^ the ESO Data Collaborative is a national, deidentified, prehospital electronic health record dataset in which participating agencies voluntarily submit data for research and benchmarking purposes. The dataset is compliant with National EMS Information System 3.4 standards, and captures dispatch-, demographic-, and encounter-specific variables.^26^ For the 2022 year of analysis, the dataset included 12,803,154 records from 2,705 participating agencies. The study was deemed exempt from review by the Yale University Institutional Review Board.

### Study Population

All 9–1-1 responses between January 1–December 31, 2022, in which the responding ambulance was first on scene and the patient was alive were included. We excluded records where the disposition was “dead on scene,” the primary impression was “obvious death,” or the location type was “morgue.” As the range of disability varies, we also excluded patients who may be deemed unable to refuse care, including those who had a developmental or psychological disability, were unconscious, or had an Alert, Voice, Pain, Unresponsiveness (AVPU) scale score of 1, indicating they were unresponsive. Similarly, patients < 18 years of age were excluded as their ability to consent to care independent of a guardian varies by age, state, and condition. We also excluded records with covariates that could influence time on scene or non-transport such as sex, race, ethnicity, agency, location, chief complaint, level of service, and medical/trauma designation data.

### Data Management

**Figure 1:**
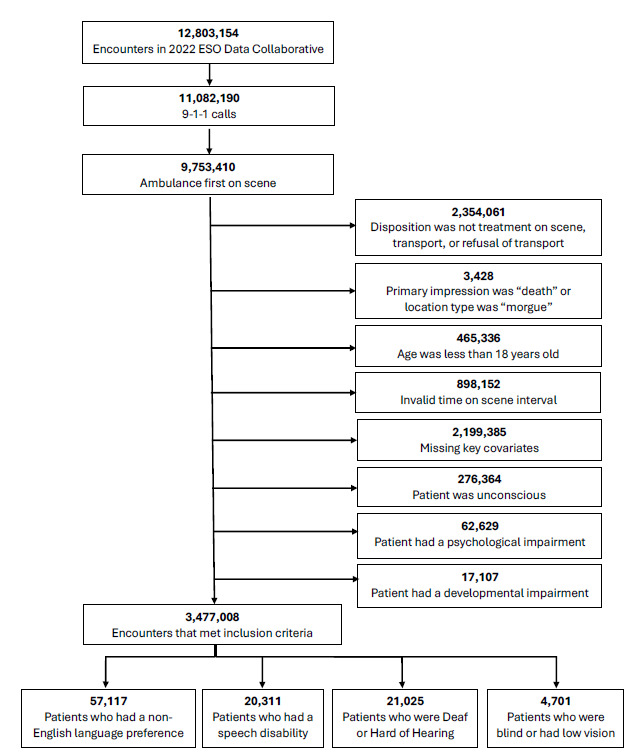
Analytic sample flow diagram in a study of EMS on-scene time with patient/paramedic communication barriers.

To capture similar response categories, we collapsed responses for three variables: disposition; level of service; and encounter location. Dispositions were classified into mutually exclusive categories of treatment on scene, transport, and non-transport based on the patient’s status at the end of the encounter. We excluded encounters with dispositions not collapsible within these three categories (Figure 1). For the level of service variable, we reclassified discrepancies between the “level of care” and “level of service” variables by selecting the lower of the two options to account for the training level of the crew or available resources on the ambulance. For example, if the level of care was categorized as Advanced Life Support (ALS) and the level of service was categorized as Basic Life Support (BLS), the final determination was BLS given that was the highest level at which the crew could perform. Location was reclassified into home or residence, medical (for encounters where the location was a medical setting), and public (for encounters that occurred in publicly accessible areas). Classification tables for the disposition, level of service, and encounter location are available in the Supplemental Appendix.

### Outcome Measures and Covariates

The primary outcomes of interest were time on scene and non-transport. We defined time on scene as the interval between “at patient time” and “depart scene time” data fields, rounded to the nearest minute. We excluded records that did not have a valid time interval, defined as < 0 minutes or > 240 minutes, as these calls were more likely to be atypical, complex, or multi-patient responses.

Independent variables of interest included time-on-scene intervals, age, sex, race/ethnicity, level of service, location type, agency type, type of encounter, chief complaint system, and communication barriers. To account for extreme values, we winsorized age at the 99^th^ percentile, effectively capping age at 95 years. For race and ethnicity calculations, an “or” statement was used to classify patients as Hispanic or Latino if the race or ethnicity variables were marked as such, consistent with prior ESO literature approaches.^22^ Those with communication barriers were inclusive of encounters in which a “language barrier,” “hearing impairment,” “speech impairment,” or “vision impairment” were identified and documented by the responding EMS clinician. It is important to note that the language used surrounding communication barriers in the ESO dataset may not necessarily reflect the language that patients and first responders may use. For example, the dataset uses the term “hearing impairment.” While some people with hearing loss may use this term, it does not necessarily reflect the language that may be used by those who are deaf or hard of hearing, and may be found offensive by others.^27^ Although these were the terms used in the dataset, we refer to these conditions using more culturally appropriate language.

### Statistical Analysis

We calculated descriptive statistics at the patient and encounter level. The mean time on scene and standard deviation were then calculated for encounters with and without documented communication barriers. Mean was chosen over median given the normal distribution of the time on scene data. We used logistic regression models to characterize the relationship between communication barriers and non-transport, adjusting for the aforementioned covariates. Race, agency type, and chief complaint system were treated as binary variables and were compared to not having the variable documented. For example, a biracial patient who had two races documented would be captured within each of the indicated races. All analyses were completed using R v4.2 (R Foundation for Statistical Computing, Vienna, Austria).

## RESULTS

The analytic sample consisted of 3,477,008 encounters (Table 1). Most responses were medical (80.6%) compared to trauma (16.6%), at the ALS level (72.1%) compared to BLS (27.9%), and to a patient’s home or residence (64.2%) compared to medical (15.4%) or public (20.4%) locations. The sample was predominantly female (53.5%) and White (71.7%), and the mean age (SD) was 60.2 (20.6). Of the > 3 million encounters, 57,117 (1.6%) were patients with an identified non-English language preference, 20,311 (0.6%) had an identified speech disability, 21,025 (0.6%) were deaf or hard of hearing, and 4,701 (0.1%) were blind or had low vision.

### Time on Scene

**Figure 2:**
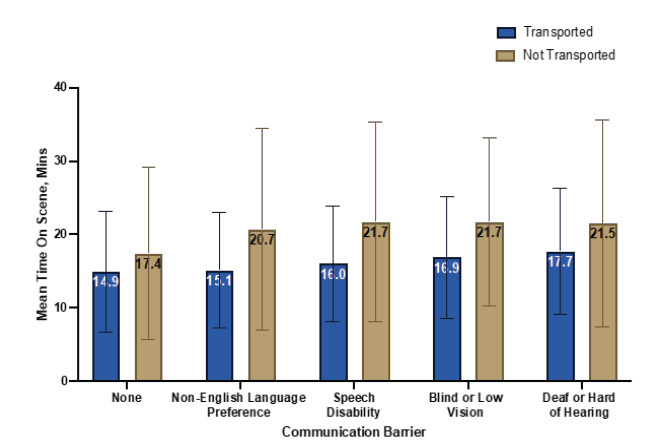
Time spent on scene by emergency medical service responders by documented communication barrier.

Prior to transport, EMS clinicians spent a mean (SD) of 15.9 (8.1) minutes of on-scene time with patients who had communication barriers compared to 14.9 (8.2) minutes with patients who had no communication barriers. Time on scene varied by communication barrier (Figure 2). More specifically, EMS clinicians spent a mean of 15.1 (7.9) minutes with patients who had a non-English language preference, 16.0 (7.9) minutes with patients who had speech disabilities, 17.7 (8.6) minutes with patients who were deaf or hard of hearing, and 16.9 (8.3) minutes with patients who were blind or had low vision.

Among all encounters, EMS clinicians spent more time on scene caring for patients who were not transported compared to those who were transported. When a communication barrier was present, EMS clinicians spent a mean of 21.0 (13.7) minutes on scene with patients who were not transported compared to 15.9 (8.1) minutes with patients who were transported for definitive care (*P* < .001). More specifically, EMS clinicians spent 20.7 (13.8) minutes with patients who had documented non-English language preference, 21.7 (13.6) minutes with patients who had documented speech disabilities, 21.5 (14.1) minutes with patients who were deaf or hard of hearing, and 21.7 (11.4) minutes with patients who were blind or had low vision. These differences were statistically significant (*P* < .001).

### Non-Transport

**Figure 3:**
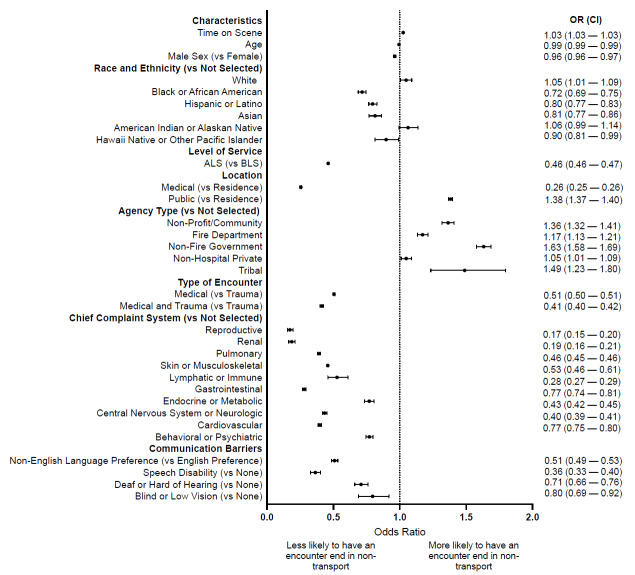
Associations between non-transport and encounter and patient-level characteristics Table 1. Sample characteristics by communication barrier, N (%) in a study of EMS on-scene time with patient/paramedic communication barriers. *OR*, odds ratio; *CI*, confidence interval; *ALS*, Advanced Life Support; *BLS*, Basic Life Support.

A total of 233,084 (6.7%) encounters resulted in non-transport. More specifically, 2,048/57,117 (3.6%) encounters with a documented non-English language preference, 384/20,311 (1.9%) encounters with a documented speech disability, 843/21,025 (4.0%) encounters with a patient who was deaf or hard of hearing, and 205/4,701 (4.4%) encounters with a patient who was blind or had low vision ended in non-transport. Additionally, patients with communication barriers, including non-English language preference (odds ratio [OR] 0.51, confidence interval [CI] 0.49–0.53, *P* < .001), speech disabilities (OR 0.36, CI 0.33–0.40, *P* < .001), who were deaf or hard of hearing (OR 0.71, CI 0.66–0.76, *P* < .001), or were blind or had low vision (OR 0.80, CI 0.69–0.92, *P* < .001) were less likely to have the encounter end in non-transport (Figure 3).

## DISCUSSION

In this analysis of > 3 million EMS records, we identified four major findings. First, in a nationally representative sample, approximately 3% of prehospital encounters had evidence of a communication barrier. Second, prior to transport, average on-scene time was approximately 7% greater for encounters with a documented communication barrier compared to those without a communication barrier. Third, when communication barriers were present, EMS clinicians spent approximately 32% more time on encounters that ended in non-transport compared to encounters where the patient was transported for definitive care. Fourth, encounters with identified communication barriers were less likely to result in non-transport. These findings provide important and actionable information for EMS leaders, agencies, and policymakers.

Compared to national estimates,^2^–^5^ the prevalence of communication barriers among prehospital encounters was low. There are several possible explanations for this finding. If a communication barrier is mild, it may be under-reported by the patient or undocumented by the clinician, especially if the EMS clinician can navigate around the barrier by implementing other communication strategies and techniques. Since the degree of impact on communication is not defined for it to be reported, each clinician must use individual judgment regarding the magnitude of a barrier that warrants documentation. The low prevalence of communication barriers in this sample may also be reflective of larger barriers faced by this population. Patients with communication barriers have reported hesitancy and uncertainty about contacting 9-1-1, difficulty communicating with dispatchers, and trouble understanding instructions issued by dispatchers,^6^,^7^,^14^ all of which can lead to lower engagement with EMS. With an aging and increasingly more diverse national population, it is likely that the prevalence of these communication barriers and, therefore, the relevance of these findings will increase.

Emergency medical services clinicians spend more time on scene with patients who have communication barriers and are more likely to transport them for definitive care. Agencies that receive federal funds are required to evaluate the need for language services and implement solutions accordingly.^28^ Patients might benefit from EMS clinicians being equipped with training and resources to improve and expedite care. Multiple strategies may be deployed in the prehospital setting to overcome communication barriers that address ambulance design and clinician practices. Regarding system-level changes to ambulance design, ambulances could be engineered to reduce background noise in patient care areas and incorporate assistive listening devices, magnifiers, and mobile interpreting software. The EMS clinicians can also employ individual practice strategies to improve communication, including minimizing disruptions such as background noise, using hearing amplifiers/assistive listening devices, moving to well-lit areas, writing down or drawing instructions for patients, learning basic instructions and maintaining refusal forms in commonly spoken languages, verbalizing actions to patients with decreased visual acuity, and incorporating available family members to advise the crew regarding accommodation strategies.

Training programs in EMS have developed workshops aimed at improving communication with patients who have disabilities impacting speech or are hard of hearing.^29^ Similarly, other programs have piloted resources such as communication boards to target patients who are hard of hearing or speak another language.^30^ When interacting with patients with a non-English language preference, EMS clinicians report preferring ad hoc interpreters, such as bystanders or children, and translation apps over formal telephonic interpretation due to difficulties accessing timely telephonic interpretation.^14^ However, this may be problematic as family members, friends, and especially minor children are prone to omissions, additions, and substitutions.^31^,^32^ Given the preference for on-scene interpretation, EMS agencies may reduce on-scene time of encounters by recruiting and training bilingual staff. These measures can help reduce the time on scene and ensure that patients with communication barriers receive appropriate and timely care.

Responders spend more time on scene with patients who ultimately refuse transport compared to those who are transported for definitive care. Proper patient education and adherence to documentation may explain the increased time on scene for this patient population. The EMS clinicians may take more time to appropriately ensure the patient is competent to decline further care, understands the implications of their decision, and signs appropriate documentation. Depending on the population call density and geography, five additional minutes (32% more) of on-scene time may have an impact on overall system productivity and unit hour use. As EMS systems strive to care for more complex patient populations, integrating specific communication training programs and assistive tools, such as hearing amplifiers and multilingual support systems, may alleviate some prehospital care challenges.

Our analyses also identified that encounters with documented communication barriers were less likely to result in non-transport. Many EMS protocols consider the presence of communication barriers as high-risk refusals, and EMS clinicians may be more apt to strongly recommend or be required by protocol to transport.^33^ There may also be contributory social and cultural factors (eg, patients with a non-English language preference may feel less comfortable refusing care or may have difficulty conveying refusal). In addition, there may be scenarios in which patients with disabilities that impact vision, speech, or hearing, may present a challenge for EMS clinicians to assess whether they have the capacity to refuse care as some presentations may overlap with emergent presentations of illness. Understanding that communication barriers lead to fewer non-transport instances can inform EMS protocols and encourage the development of standardized approaches to manage high-risk refusals effectively.

Central to this research question, EMS practice often varies based on clinical condition and setting that dictates whether shorter or longer on-scene times are more beneficial to patient outcomes.^6^ Research assessing the safety of non-transport has evolved over the years with studies focusing on and finding mixed results for paramedic-initiated non-transport.^34^–^37^ The success of these encounters and decisions remains contingent on clear communication between patients and EMS clinicians. Unique challenges may persist for patients with communication barriers in this changing landscape. Future research should examine whether on-scene time impacts outcomes for patients with communication barriers. Additionally, research should explore outcomes following non-transport for patients with communication barriers. As EMS agencies move away from operational metrics, such as response times, to performance measures focusing on the administration of appropriate treatment in a timely manner, more research is needed to determine whether disparities exist for patients with communication barriers.^38^

## LIMITATIONS

The ESO Data Collaborative is a retrospective database with several limitations. Communication barriers were documented as a binary variable in the dataset. Therefore, we were unable to describe the degree of impact of the disability or the fluency with which the patient or EMS responder spoke a common language. It is also unclear whether the identified disability was perceived by the EMS clinician or disclosed by the patient during the encounter, and causation cannot be ultimately inferred as this was an observational study. However, if it is likely that communication barriers with minimal impact were less likely to be reported, then it is possible that the prevalence estimates calculated are underestimates of the true prevalence, thereby further reinforcing the importance of this issue for EMS clinicians.

Although over 2,000 EMS agencies submit data for research and benchmarking purposes, the ESO database does not capture all national EMS encounters. The voluntary nature of submission may restrict generalizability as EMS agencies in urban areas or urban clusters have previously been shown to be more likely to submit their data.^39^ Lastly, there may be regional variation in protocols that dictate treatment and transportation decision-making (such as a requirement to contact medical control for high-risk refusals) that could not be captured from this dataset.

## CONCLUSION

In this nationally representative prehospital analysis, approximately 1 in 30 encounters had evidence of a barrier to communication. Encounters impacted by communication barriers were associated with a longer average time on scene compared to encounters without communication barriers. Encounters associated with communication barriers were less likely to result in non-transport. Emergency medical services clinicians may benefit from being equipped with training and resources to communicate effectively with these vulnerable populations and anticipate longer on-scene time to provide appropriate care.

## Supplementary Information









## Figures and Tables

**Table 1:** Sample characteristics by communication barrier, N (%) in a study of EMS on-scene time with patient/paramedic communication barriers.

Characteristic	Total	Deaf or hard of hearing	Non-English language preference	Speech disability	Blind or low vision
Total	3,477,008	21,025	57,117	20,311	4,701
Age (Mean years, SD)	60.2 (20.6)	80.9 (14.9)	57.4 (21.5)	66.6 (16.7)	68.9 (18.1)
Sex					
Male	1,615,469 (46.5)	10,231 (48.7)	27,596 (48.3)	10,837 (53.4)	2,293 (48.8)
Female	1,861,539 (53.5)	10,794 (51.3)	29,521 (51.7)	9,474 (46.6)	2,408 (51.2)
Race/Ethnicity					
White	2,492,382 (71.7)	18,271 (86.9)	8,538 (14.9)	13,856 (68.2)	3,097 (65.9)
Black	701,777 (20.2)	1,732 (8.2)	4,624 (8.1)	4,816 (23.7)	1,264 (26.9)
Hispanic or Latino	237,473 (6.8)	704 (3.3)	36,068 (63.1)	1,265 (6.2)	277 (5.9)
Asian	36,302 (1.0)	256 (1.2)	7,206 (12.6)	298 (1.5)	41 (0.9)
American Indian/Alaskan Native	15,654 (0.5)	71 (0.3)	442 (0.8)	95 (0.5)	20 (0.4)
Hawaii Native or other Pacific Islander	7,728 (0.2)	37 (0.2)	768 (1.3)	56 (0.3)	8 (0.2)
Level of Service					
ALS	2,506,948 (72.1)	14,726 (70.0)	40,074 (70.2)	15,094 (74.3)	3,254 (69.2)
BLS	970,060 (27.9)	6,299 (30.0)	17,043 (29.8)	5,217 (25.7)	1,447 (30.8)
Agency Type					
Non-profit community	1,455,155 (41.9)	8,705 (41.4)	21,195 (37.1)	8,687 (42.8)	1,933 (41.1)
Fire department	873,752 (25.1)	5,197 (24.7)	19,734 (34.6)	4,953 (24.4)	969 (20.6)
Non-fire government	773,205 (22.2)	4,551 (21.6)	9,609 (16.8)	4,499 (22.2)	1,190 (25.3)
Hospital	77,685 (2.2)	590 (2.8)	765 (1.3)	436 (2.1)	97 (2.1)
Private	295,717 (8.5)	1,965 (9.3)	5,753 (10.1)	1,718 (8.5)	508 (10.8)
Tribal	1,494 (0.0)	17 (0.1)	61 (0.1)	18 (0.1)	4 (0.1)
Location Type					
Home/Residence	2,232,190 (64.2)	14,290 (68.0)	33,521 (58.7)	12,030 (59.2)	3,177 (67.6)
Medical facility	536,836 (15.4)	5,382 (25.6)	6,099 (10.7)	6,137 (30.2)	1,120 (23.8)
Public space	707,982 (20.4)	1,353 (6.4)	17,497 (30.6)	2,144 (10.6)	404 (8.6)
Type of encounter					
Medical	2,801,802 (80.6)	15,501 (73.7)	40,740 (71.3)	17,585 (86.6)	3,839 (81.7)
Trauma	578,219 (16.6)	4,345 (20.7)	14,656 (25.7)	1,877 (9.2)	651 (13.8)
Medical and trauma	96,987 (2.8)	1,179 (5.6)	1,721 (3.0)	849 (4.2)	211 (4.5)

*ALS*, Advanced Life Support; *BLS*, Basic Life Support.
