# Supplementary material for: Emergency Medical Services Time on Scene and Non-Transport: Role of Communication Barriers
Source: West J Emerg Med. 2025 Aug 20;26(5):1265–73. doi: 10.5811/westjem.41212 (PMC12591645; doi:10.5811/westjem.41212)
Supplement: Supplementary file 1 [file wjem-26-1265-s001.docx]

**Table A1.** Categorization of the “disposition” variable to determine treatment on scene, transport, non-transport, or exclusion from study

| **Variable** | **Determination** |
| --- | --- |
| Transported no lights/siren | Transport |
| Cancelled (prior to arrival at scene) | Exclude |
| Cancelled on scene/no patient found | Exclude |
| Cancelled (no patient contact) | Exclude |
| Patient evaluated, no treatment/transport required | Non-transport |
| Transported lights/siren | Transport |
| Patient treated, transferred care to another ems professional/unit | Exclude |
| Patient treated, released (ama) | Treatment on Scene |
| Patient treated, released (per protocol) | Treatment on Scene |
| Patient refused evaluation/care (without transport) | Non-transport |
| Standby - public safety, fire, or ems operational support provided | Exclude |
| Assist, agency | Exclude |
| Assist, unit | Exclude |
| Assist, public | Exclude |
| Patient dead on scene - no resuscitation attempted (without transport) | Exclude |
| Patient treated, transported by law enforcement | Exclude |
| Patient dead on scene - resuscitation attempted (without transport) | Exclude |
| Transported lights/siren, downgraded | Transport |
| Wheelchair transport | Exclude |
| Transported no lights/siren, upgraded | Transport |
| Standby - no service or support provided | Exclude |
| Patient treated, transported by private vehicle | Exclude |
| Patient dead on scene - no resuscitation attempted (with transport) | Exclude |
| Transport non-patient, organs, etc.. | Exclude |
| Patient treated, transferred care to a non-ems provider on scene | Exclude |
| Patient dead on scene - resuscitation attempted (with transport) | Exclude |
| Patient refused evaluation/care (with transport) | Transport |
| Patient treated, transferred care to a telehealth provider | Exclude |
| No treatment, no transport | Non-transport |
| False alarm (no incident occurred) | Exclude |
| Disregarded enroute | Exclude |
| Treatment, no transport | Treatment on Scene |
| No patient found | Exclude |
| Call cancelled | Exclude |
| Personnel aiding in transport | Exclude |
| Dead on scene, no transport | Exclude |
| Standby | Exclude |
