# Supplementary material for: Emergency Medical Services Time on Scene and Non-Transport: Role of Communication Barriers
Source: West J Emerg Med. 2025 Aug 20;26(5):1265–73. doi: 10.5811/westjem.41212 (PMC12591645; doi:10.5811/westjem.41212)
Supplement: Supplementary file 2 [file wjem-26-1265-s002.docx]

**Table A2**. Categorization of unit and level of service variables to determine level of service

| **Level of Service** | **Unit Level of Service** | **Determination** |
| --- | --- | --- |
| "" | "als-aemt" | ALS |
| "" | "als-community paramedicine" | ALS |
| "" | "als-intermediate" | ALS |
| "" | "als-nurse" | ALS |
| "" | "als-paramedic" | ALS |
| "" | "als-physician" | ALS |
| "" | "bls-aemt" | BLS |
| "" | "bls-basic /emt" | BLS |
| "" | "bls-community paramedicine" | BLS |
| "" | "bls-first responder/emr" | BLS |
| "" | "bls-intermediate" | BLS |
| "" | "nys emt - critical care" | ALS |
| "" | "specialty critical care" | Exclude |
| "advanced life support" | "" | ALS |
| "advanced life support" | "als-aemt" | ALS |
| "advanced life support" | "als-community paramedicine" | ALS |
| "advanced life support" | "als-intermediate" | ALS |
| "advanced life support" | "als-nurse" | ALS |
| "advanced life support" | "als-paramedic" | ALS |
| "advanced life support" | "als-physician" | ALS |
| "advanced life support" | "bls-aemt" | BLS |
| "advanced life support" | "bls-basic /emt" | BLS |
| "advanced life support" | "bls-community paramedicine" | BLS |
| "advanced life support" | "bls-first responder/emr" | BLS |
| "advanced life support" | "bls-intermediate" | BLS |
| "advanced life support" | "nys emt - critical care" | ALS |
| "advanced life support" | "specialty critical care" | ALS |
| "als 2" | "" | ALS |
| "als 2" | "als-aemt" | ALS |
| "als 2" | "als-community paramedicine" | ALS |
| "als 2" | "als-intermediate" | ALS |
| "als 2" | "als-nurse" | ALS |
| "als 2" | "als-paramedic" | ALS |
| "als 2" | "als-physician" | ALS |
| "als 2" | "bls-aemt" | BLS |
| "als 2" | "bls-basic /emt" | BLS |
| "als 2" | "bls-first responder/emr" | BLS |
| "als 2" | "bls-intermediate" | BLS |
| "als 2" | "nys emt - critical care" | ALS |
| "als 2" | "specialty critical care" | ALS |
| "als, downgraded" | "" | ALS |
| "als, downgraded" | "als-aemt" | ALS |
| "als, downgraded" | "als-intermediate" | ALS |
| "als, downgraded" | "als-nurse" | ALS |
| "als, downgraded" | "als-paramedic" | ALS |
| "als, downgraded" | "als-physician" | ALS |
| "als, downgraded" | "bls-aemt" | BLS |
| "als, downgraded" | "bls-basic /emt" | BLS |
| "als, downgraded" | "bls-first responder/emr" | BLS |
| "als, downgraded" | "bls-intermediate" | BLS |
| "als, downgraded" | "nys emt - critical care" | BLS |
| "als, downgraded" | "specialty critical care" | BLS |
| "basic life support" | "" | BLS |
| "basic life support" | "als-aemt" | BLS |
| "basic life support" | "als-community paramedicine" | BLS |
| "basic life support" | "als-intermediate" | BLS |
| "basic life support" | "als-nurse" | BLS |
| "basic life support" | "als-paramedic" | BLS |
| "basic life support" | "als-physician" | BLS |
| "basic life support" | "bls-aemt" | BLS |
| "basic life support" | "bls-basic /emt" | BLS |
| "basic life support" | "bls-community paramedicine" | BLS |
| "basic life support" | "bls-first responder/emr" | BLS |
| "basic life support" | "bls-intermediate" | BLS |
| "basic life support" | "nys emt - critical care" | BLS |
| "basic life support" | "specialty critical care" | BLS |
| "bls, upgraded" | "" | BLS |
| "bls, upgraded" | "als-aemt" | ALS |
| "bls, upgraded" | "als-intermediate" | ALS |
| "bls, upgraded" | "als-nurse" | ALS |
| "bls, upgraded" | "als-paramedic" | ALS |
| "bls, upgraded" | "als-physician" | ALS |
| "bls, upgraded" | "bls-aemt" | BLS |
| "bls, upgraded" | "bls-basic /emt" | BLS |
| "bls, upgraded" | "bls-community paramedicine" | BLS |
| "bls, upgraded" | "bls-first responder/emr" | BLS |
| "bls, upgraded" | "bls-intermediate" | BLS |
| "bls, upgraded" | "specialty critical care" | Exclude |
| "critical care" | "" | ALS |
| "critical care" | "als-aemt" | ALS |
| "critical care" | "als-community paramedicine" | ALS |
| "critical care" | "als-intermediate" | ALS |
| "critical care" | "als-nurse" | ALS |
| "critical care" | "als-paramedic" | ALS |
| "critical care" | "als-physician" | ALS |
| "critical care" | "bls-aemt" | BLS |
| "critical care" | "bls-basic /emt" | BLS |
| "critical care" | "bls-first responder/emr" | BLS |
| "critical care" | "bls-intermediate" | BLS |
| "critical care" | "nys emt - critical care" | ALS |
| "critical care" | "specialty critical care" | ALS |
| "first responder" | "als-aemt" | BLS |
| "first responder" | "als-community paramedicine" | BLS |
| "first responder" | "als-intermediate" | BLS |
| "first responder" | "als-paramedic" | BLS |
| "first responder" | "bls-aemt" | BLS |
| "first responder" | "bls-basic /emt" | BLS |
| "first responder" | "bls-first responder/emr" | BLS |
| "other" | "" | Exclude |
| "other" | "als-aemt" | ALS |
| "other" | "als-community paramedicine" | ALS |
| "other" | "als-intermediate" | ALS |
| "other" | "als-nurse" | ALS |
| "other" | "als-paramedic" | ALS |
| "other" | "bls-aemt" | BLS |
| "other" | "bls-basic /emt" | BLS |
| "other" | "bls-community paramedicine" | BLS |
| "other" | "bls-first responder/emr" | BLS |
| "other" | "bls-intermediate" | BLS |
| "other" | "specialty critical care" | ALS |
| "pediatric advanced life support" | "als-nurse" | ALS |
| "pediatric advanced life support" | "als-paramedic" | ALS |
| "pediatric advanced life support" | "als-physician" | ALS |
| "pediatric advanced life support" | "bls-basic /emt" | BLS |
| "pediatric advanced life support" | "specialty critical care" | ALS |
| "specialty care transport" | "" | Exclude |
| "specialty care transport" | "als-aemt" | Exclude |
| "specialty care transport" | "als-community paramedicine" | Exclude |
| "specialty care transport" | "als-intermediate" | Exclude |
| "specialty care transport" | "als-nurse" | Exclude |
| "specialty care transport" | "als-paramedic" | Exclude |
| "specialty care transport" | "als-physician" | Exclude |
| "specialty care transport" | "bls-aemt" | Exclude |
| "specialty care transport" | "bls-basic /emt" | Exclude |
| "specialty care transport" | "bls-first responder/emr" | Exclude |
| "specialty care transport" | "bls-intermediate" | Exclude |
| "specialty care transport" | "specialty critical care" | Exclude |
