# Supplementary material for: Emergency Medical Services Time on Scene and Non-Transport: Role of Communication Barriers
Source: West J Emerg Med. 2025 Aug 20;26(5):1265–73. doi: 10.5811/westjem.41212 (PMC12591645; doi:10.5811/westjem.41212)
Supplement: Supplementary file 3 [file wjem-26-1265-s003.docx]

**Table A3.** Categorization of the “location type” variable to determine encounter location

| **Variable** | **Determination** |
| --- | --- |
| Airport | Public |
| Assisted living center | Medical |
| Cinema / theater | Public |
| Doctor's office / clinic | Medical |
| EMS provider (ground) | Medical |
| Freestanding er | Medical |
| Hospital | Medical |
| Lake, river, ocean | Public |
| Mine/quarry | Public |
| Other recurring care center | Medical |
| Place of recreation/sport | Public |
| Qualified health care partner | Medical |
| Religious institution | Public |
| School | Public |
| Train station | Public |
| Alternate care site | Medical |
| Beach | Public |
| Diagnostic services | Medical |
| Drug and/or alcohol rehabilitation facility | Medical |
| Farm | Public |
| Healthcare practitioner | Medical |
| Hospital (other location) | Medical |
| Mental health facility | Medical |
| Morgue | Exclude |
| Other specified place | Public |
| Police/jail | Public |
| Recreation area | Public |
| Residential institution | Home |
| Skilled nursing facility | Medical |
| Urgent care | Medical |
| Amusement park | Public |
| Bus station | Public |
| Dialysis | Medical |
| EMS provider (air) | Medical |
| Federally qualified health center | Medical |
| Home/residence | Home |
| Industrial place | Public |
| Military base | Public |
| Nursing home | Medical |
| Place of business | Public |
| Public building | Public |
| Rehabilitation center | Medical |
| Rural health clinic | Medical |
| Street or highway | Public |
| Wilderness area | Public |
